# Phenolic Constituents with Antioxidative, Tyrosinase Inhibitory and Anti-aging Activities from *Dendrobium loddigesii* Rolfe

**DOI:** 10.1007/s13659-019-00219-y

**Published:** 2019-10-19

**Authors:** Rui-Jing Ma, Liu Yang, Xue Bai, Jin-Yu Li, Ming-Yan Yuan, Ya-Qin Wang, Yong Xie, Jiang-Miao Hu, Jun Zhou

**Affiliations:** 1 State Key Laboratory of Phytochemistry and Plant Resources in West China, Kunming Institute of Botany, Chinese Academy of Sciences, Kunming, 650201 People’s Republic of China; 2 College of Pharmacy, Guilin Medical University, Guilin, 541004 People’s Republic of China; 3 R & D Center of Dr. Plant, Kunming Institute of Botany, Chinese Academy of Sciences, Kunming, 650201 People’s Republic of China

**Keywords:** *Dendrobium loddigesii*, Phenolic constituents, Antioxidative, Tyrosinase inhibitory, Anti-aging

## Abstract

**Abstract:**

Aqueous ethanol extracts of powdered stems of *Dendrobium loddigesii* afforded three new phenolics including *threo-*7-*O-*ethyl*-*9-*O-*(4-hydroxyphenyl)propionyl-guaiacylglycerol (**1**), (*R*)-4,5,4ʹ-trihydroxy-3,3ʹ,α-trimethoxybibenzyl (**2**) and (*S*)-5,5′,7-trihydroxy-3′,4′-dimethoxyflavanone (**3**), together with eleven known analogues. Their structures were determined by extensive spectroscopic analysis. To identify natural antioxidants, whitening, and anti-aging agents, the abilities of these phenolics were assessed to scavenge the 1,2-diphenyl-2-picrylhydrazyl (DPPH) radical, their abilities to inhibit tyrosinase production, and their abilities to stimulate collagen production by human dermal fibroblasts-adult (HDFa) assay. It was found that compounds **1**, **4**–**8**, **13** and **14** exhibited significant DPPH radical scavenging activities, compound **10** exhibited tyrosinase inhibitory activity (IC_50_ 37.904 μg/mL), and compound **9** showed significant collagen production with an EC_50_ value of 3.182 μg/mL. These results suggest that phenolic constituents from *D. loddigesii* may be candidate antioxidants, skin-whitening and/or anti-aging agents.

**Graphic Abstract:**

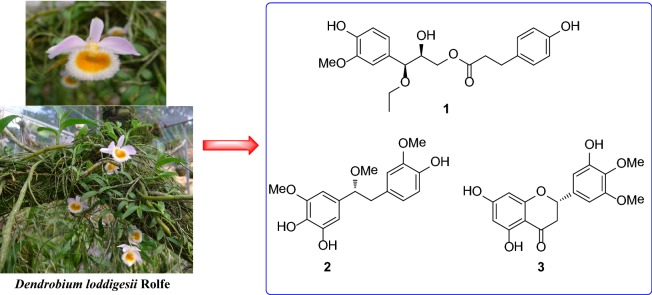

**Electronic supplementary material:**

The online version of this article (10.1007/s13659-019-00219-y) contains supplementary material, which is available to authorized users.

## Introduction

The genus *Dendrobium* (Orchidaceae) contains approximately 1500 species globally, of which about 80 species grow in China [1]. The stems of several plants in this genus are known as “Shi-Hu”, which have been used for thousands of years as both traditional Chinese medicine and folk remedies for the treatment chronic atrophic gastritis, skin aging, fever, cardiovascular disease and a tonic for promoting the production of body fluid [2]. Previous studies on this genus led to the isolation of a series of polysaccharides, phenolic compounds, alkaloids, and sesquiterpenoids [1, 3–5], some of which possess various bioactivities including anti-inflammatory [6], antimicrobial [2], antioxidant [7], antitumor [8], antiplatelet aggregation [9], immunomodulatory [10], and against influenza A activities [11].

**Fig. 1:**
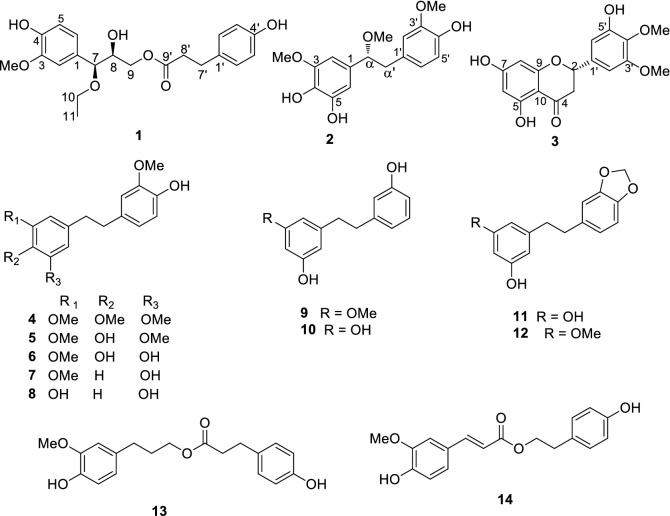
Chemical structures of compounds **1**–**14**

*Dendrobium loddigesii*, a perennial epiphytic herb, is widely distributed in the southwestern area of China, such as Guangxi, Guizhou, and Yunnan Provinces [12]. Its stem has been applied in folk medicine to treat gastrosis, fever, and dizziness [13]. In continuing the search for structurally diverse and biologically active natural products from this genus [1, 5, 14–17], an in-depth investigation of pharmacologically active constituents from this plant species was performed herein. As a result, three new phenolic compounds (**1**–**3**) as well as eleven known ones (Fig. 1) were isolated from an 80% ethanolic extract of the stem of *D. loddigesii*. The isolation, structure elucidation, and biological evaluation of these compounds are presented herein.

## Results and Discussion

**Fig. 2:**
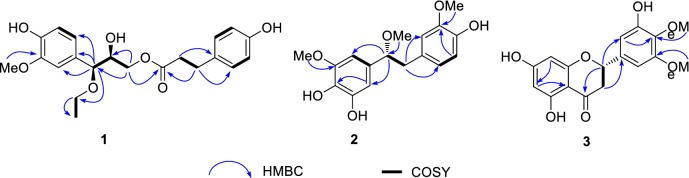
The key HMBC and COSY correlations of compounds **1**–**3**

Compound **1**, obtained as white solid, gave a molecular formula of C_21_H_26_O_7_ as determined by the (−)-HRESIMS ion at *m/z* 389.1602 [M−H]^−^ (calcd for C_21_H_25_O_7_, 389.1606) with nine degrees of unsaturation. The ^1^H NMR spectrum of **1** contains seven aromatic protons at *δ*_H_ 6.85 (1H, d, *J* = 1.7 Hz), 6.75 (1H, d, *J* = 8.0 Hz), 6.68 (1H, dd, *J* = 8.0, 1.7 Hz), 7.01 (2H, d, *J* = 8.5 Hz) and 6.68 (2H, d, *J* = 8.5 Hz), suggesting the presence of a 1,3,4-trisubstituted benzene ring and a 1,4-disubstituted benzene ring. Its ^13^C NMR spectrum exhibited 21 carbon resonances including two methyl (one methoxy), four aliphatic methylene, nine methine (two *sp*^3^, seven *sp*^2^), and six quaternary carbons (one carbonyl, five olefinic including three oxygenated). The HMBC correlations (Fig. 2) of H-7/C-1 (*δ*_C_ 131.6), C-2 (*δ*_C_ 111.7), C-6 (*δ*_C_ 121.4), C-8 (*δ*_C_ 74.2) and C-10 (*δ*_C_ 65.3); H-9/C-7 (*δ*_C_ 83.8) and C-8 (*δ*_C_ 74.2); H-10/C-11 (*δ*_C_ 15.6); 3-OMe (*δ*_H_ 3.81)/C-3 (*δ*_C_ 149.1), together with correlations of H-7/H-8/H_2_-9 and H_2_-10/H_3_-11 from ^1^H–^1^H COSY spectrum (Fig. 2) indicated the presence of 7-*O*-ethylguaiacylglycerol [18]. It has been reported that *J*_7,8_ was about 5 Hz for the erythro isomer and 7 Hz for the threo isomer in the cases of syringoyl-glycerols and guaiacylglycerol derivatives. Thus, compound **1** was considered to be the threo isomer with *J*_7,8_ (6.5 Hz) [18]. The HMBC correlations of H-7ʹ (*δ*_H_ 2.79, t, *J* = 7.5 Hz)/C-8ʹ (*δ*_C_ 37.1), C-1ʹ (*δ*_C_ 132.7), C-2ʹ, 6ʹ (*δ*_C_ 130.2) and C-9ʹ (*δ*_C_ 174.6), H-8ʹ (*δ*_H_ 2.59, t, *J* = 7.5 Hz)/C-7ʹ (*δ*_C_ 31.0), C-1ʹ, and C-9ʹ, along with COSY cross-peaks of H-8ʹ/H-7ʹ indicated the presence of *p*-hydroxycoumaric acid [19]. On the basis of the evidences described above, **1** was proposed to have a 7-*O*-ethylguaiacylglycerol moiety and a *p*-hydroxycoumaric acid via an ester linkage. The HMBC correlation from H-9 to C-9ʹ suggested the ester linkage was between C-9 and C-9ʹ. Thus, the structure of **1** was determined as shown.

(*R*)-4,5,4ʹ-Trihydroxy-3,3ʹ,α-trimethoxybibenzyl (**2**) was obtained as white solid. The HRESIMS spectrum of **2** displayed a quasimolecular ion peak at *m/z* 319.1180 [M−H]^−^ (calcd. for C_17_H_19_O_6_, 319.1187) with 8 degrees of unsaturation. The ^1^H NMR spectrum of **2** showed three methoxyl groups at *δ*_H_ 3.75 (3H, s), 3.70 (3H, s) and 3.17 (3H, s); one oxygenated methine proton at *δ*_H_ 4.13 (1H, t, *J* = 6.8 Hz, H-α); two methylene signals at *δ*_H_ 2.73 (1H, dd, *J* = 13.5, 6.9 Hz) and 2.96 (1H, dd, *J* = 13.5, 6.9 Hz); and five aromatic protons, appearing as a 1,3,4,5-tetrasubstituted aromatic ring at *δ*_H_ 6.28 (1H, d, *J* = 1.8 Hz) and 6.34 (1H, d, *J* = 1.8 Hz), and a 1,3,4-trisubstituted aromatic ring at *δ*_H_ 6.49 (1H, d, *J* = 2.0 Hz), 6.62 (1H, d, *J* = 8.0 Hz) and 6.52 (1H, dd, *J* = 8.0, 2.0 Hz). The ^13^CNMR and DEPT spectra of **2** showed three oxymethyls, one methylene, one oxygenated methine and 12 aromatic carbons (five oxygenated). Comparison of its NMR data (Table 1) with those of dendrocandin C [20] showed great similarities except for the presence of one more methoxyl group, which was located at C-3ʹ by the HMBC correlations from 3ʹ-OMe and H-5ʹ to C-3ʹ (*δ*_C_ 148.4). In addition, the multiple HMBC interactions (Fig. 2) of 3-OMe and H-2/C-3 (*δ*_C_ 149.5); α-OMe/C-α (*δ*_C_ 86.9) suggested the other methoxyl groups at C-3, and C-α, respectively. The absolute configuration at C-α was determined as *R* on the basis of the negative optical rotation ($\left[ \alpha \right]_{{\text{D}}}^{26}$ –12.46), similar to aphyllal D $\left[ \alpha \right]_{{\text{D}}}^{20}$ –20.3, MeOH) [15]. Accordingly, the structure of **2** was determined as shown.

**Table 1:** The ^1^H and ^13^C-NMR data of **1**–**3** (methanol-*d*_*4*_, *δ* in ppm, *J* in Hz)

No.	1^a^		No.	2^b^		No.	3^b^	
	*δ*_H_	*δ*_C_		*δ*_H_	*δ*_C_		*δ*_H_	*δ*_C_
1		131.6 s	1		134.6 s	1		
2	6.85 d (1.7)	111.7 d	2	6.28 d (1.8)	103.4 d	2	5.29 dd (12.7, 3.1)	80.4 d
3		149.1 s	3		149.5 s	3	3.04 dd (17.1, 12.7) 2.71 dd (17.1, 3.1)	44.2 t
4		147.5 s	4		133.4 s	4		197.4 s
5	6.75 d (8.0)	116.0 d	5		146.5 s	5		165.4 s
6	6.68 dd (8.0, 1.7)	121.4 d	6	6.34 d (1.8)	108.6 d	6	5.87 d (2.2)	97.1 d
7	4.14 d (6.5)	83.8 d	1ʹ		131.1 s	7		168.4
8	3.82 m	74.2 d	2ʹ	6.49 d (2.0)	114.4 d	8	5.91 d (2.2)	96.2 d
9	3.98 dd (11.5, 3.5) 3.78 m	66.5 t	3ʹ		148.4 s	9		164.6 s
10	3.37, 3.32 m	65.3 t	4ʹ		145.8 s	10		103.3 s
11	1.14 t (7.0)	15.6 q	5ʹ	6.62 d (8.0)	115.7 d	1ʹ		136.3 s
1ʹ		132.7 s	6ʹ	6.52 dd (8.0, 2.0)	123.1 d	2ʹ	6.62 d (2.0)	129.9 d
2ʹ	7.01 d (8.5)	130.2 d	α	4.13 t (6.9)	86.9 d	3ʹ		154.7 s
3ʹ	6.68 d (8.5)	116.2 d	α*'*	2.73 dd (13.5, 6.9) 2.96 dd (13.5, 6.9)	45.1 t	4ʹ		137.7 s
4ʹ		156.8 s	3ʹ-OMe	3.70 s	56.4 q	5ʹ		151.8 s
5ʹ	6.68 d (8.5)	116.2 d	3-OMe	3.75 s	56.5 q	6ʹ	6.61 d (2.0)	108.2 d
6ʹ	7.01 d (8.5)	130.2 d	α-OMe	3.17 s	56.7 q	3ʹ-OMe	3.83 s	56.4 q
7ʹ	2.79 t (7.5)	31.0 t				4ʹ-OMe	3.77 s	61.0 q
8ʹ	2.59 t (7.5)	37.1 t						
9*'*		174.6 s						
3-OMe	3.81 s	56.3 q						

^a1^H and ^13^C NMR data were recorded at 500 MHz and 125 MHz, respectively

^b1^H and ^13^C NMR data were recorded at 600 MHz and 150 MHz, respectively

(*S*)-5,7,5′-Trihydroxy-3′,4′-dimethoxyflavanone (**3**) was obtained as yellow amorphous powder and had the molecular C_17_H_16_O_7_ (10 indices of hydrogen deficiency) according to the (−)-HRESIMS ion at m/z 331.0819 [M – H]^−^ (calcd 331.0823). The UV absorption maxima at 206 and 294 nm indicated the presence of a flavanone [21]. The ^1^H NMR spectrum (Table 1) showed three signals in non-aromatic region placed at *δ*_H_ 5.29 (1H, dd, *J* = 12.7, 3.1, H-2), 3.04 (1H, dd, *J* = 17.1, 12.7, H-3ax), and 2.71 (1H, dd, *J* = 17.1, 3.1, H-3 eq), four aromatic protons *δ*_H_ 5.87 (1H, d, *J* = 2.2, H-6), 5.91 (1H, d, *J* = 2.2, H-8), 6.62 (1H, d, *J* = 2.0, H-2ʹ) and 6.61 (1H, d, *J* = 2.0, H-6ʹ), two methoxyl groups *δ*_H_ 3.83 (3H, s) and 3.77 (3H, s). The ^13^C NMR and DEPT spectra (Table 1) contain resonances for 17 carbons including one two methoxy, one methylene, one methine, one carbonyl carbon and 12 aromatic carbons. Comprehensive analysis of its NMR data indicated that its planar structure is closely related with that of dihydrotricin [22], except for the OH-4ʹ and OCH_3_-5ʹ resonances in dihydrotricin were transposed in **3**. This was confirmed by the HMBC cross-peaks (Fig. 2) from H-2ʹ, H-6ʹ and OCH_3_-4ʹ to C-4ʹ (*δ*_C_ 137.7), from H-6ʹ to C-5ʹ (*δ*_C_ 151.8). The absolute configuration at C-2 was postulated as being in the *S*-form on the basis of a negative specific rotation value (– 46.64, MeOH) in its optical rotation [23]. Therefore, the structure of compound **3** was thus unambiguously assigned as shown.

Eleven known compounds were identified to be crepidatin **4** [24], moscatilin **5** [25], 4,5,4′-trihydroxy-3,3′-dimethoxybibenzyl **6** [26], 4′,5-dihydroxy-3,3′-dimethoxybibenzyl **7** [27], tristin **8** [28], batatasin III **9** [27], 3,5,3′-hydroxybibenzyl **10** [29], aphyllals C **11** [15], densiflorol A **12** [30], dihydroconiferyl dihydro-*p*-coumarate **13** [31], *p*-hydroxyphenethyl *trans*-ferulate **14** [32] by spectroscopic analysis and comparing their spectral data with literature.

Phenolic compounds, are an essential part of the human diet and are known as powerful antioxidants due to their potent chain breaking action and they may contribute directly to the anti-oxidative activity [33]. The DPPH radical scavenging assay is one of the most common and relatively quick methods used for evaluating antioxidant activity. Compounds that can donate a hydrogen atom to the DPPH radical, and then gives rise to the reduced form of DPPH which will be considered as potential antioxidant agents. All compounds were evaluated for their DPPH radical scavenging activities. The present results (Table 2) exhibited that the majority of the phenolic compounds (**1**, **4**–**8**, **13** and **14**) showed significant activities with scavenging capacities ranging from 89.411 to 94.278% at 100 μg/mL.

**Table 2:** Antioxidant, tyrosinase inhibitory and collagen production activities of compounds **1**–**14**

Compound	DPPH radical scavenging % (at 100 μg/mL)	Tyrosinase inhibition % (at 100 μg/mL)	Tyrosinase inhibition IC_50_ (μg/mL)	Collagen production % (at 10 μg/mL)	Collagen production EC_50_ μg/mL
**1**	91.435	31.421	NA	− 13.987	NA
**2**	35.276	− 12.888	NA	− 3.515	NA
**3**	13.322	5.432	NA	22.974	NA
**4**	89.411	3.076	NA	22.974	NA
**5**	92.912	1.767	NA	12.747	NA
**6**	93.681	12.827	NA	33.062	NA
**7**	93.391	13.358	NA	29.157	NA
**8**	90.265	0.065	NA	24.552	NA
**9**	26.644	− 2.880	NA	78.920	3.182
**10**	33.903	75.360	37.904	16.665	NA
**11**	29.292	44.403	152.56	10.871	NA
**12**	12.041	15.249	NA	14.527	NA
**13**	91.204	23.822	NA	19.632	NA
**14**	94.278	2.618	NA	16.834	NA
Trolox^a^	96.089				
Kojic acid^b^		57.395	8.023		
TGF-β^c^				66.290	

*NA* not active

^a^Positive control used for DPPH radical scavenging assay at the concentration of 25 μg/mL

^b^Positive control used for anti-tyrosinase assay at the concentration of 10 μg/mL

^c^Positive control used for collagen production assay at the concentration of 0.01 μg/mL

On the other hand, tyrosinase is the copper containing enzyme and plays a critical role in controlling melanin biosynthesis pathway in melanocytes [34]. Therefore, tyrosinase inhibitors became important constituents of cosmetics or as medicinal products for hyperpigmentation and developing skin whitening agents. In the present study, all the isolates were evaluated for their tyrosinase inhibitory activity (Table 2). Kojic acid, a purported skin-lightening agent, was used as a positive control. 3,5,3′-hydroxybibenzyl (**10**) revealed a significant inhibitory activity with an IC_50_ value of 37.904 μg/mL. Aphyllals C (**11**) showed moderate inhibition (IC_50_, 152.56 μg/mL). All remaining compounds were inactive at concentrations up to 200 μg/mL. In this study, it can be concluded that compounds **10** and **11** can be potential candidate for the treatment of melanin biosynthesis related skin diseases.

Considering that this species medicinally used for skin aging, since, collagen is critical for skin strength and elasticity, and its degradation leads to wrinkles that accompany aging [35]. Hence, all the compounds were also purposely evaluated for their effects on collagen production in HDFa. The results (Table 2) showed that compound **9** significant stimulation HDFa collagen production activity (EC_50_ 3.182 μg/mL). Compounds **6** and **7** showed weaker activities, with collagen production of 33.062% and 29.157% at 10 μg/mL, respectively. The present results not only supported the ethnopharmacological usage of *D. loddigesii* but also provided a reliable structure template for developing collagen deficiency associated diseases such as burn and ulcer.

## Experimental

### General Experimental Procedures

Optical rotation was obtained on a JASCO P-1020 digital polarimeter (Horiba, Tokyo, Japan). UV spectra were measured using a Shimadzu UV-2401 PC spectrophotometer (Shimadzu, Kyoto, Japan). IR spectra were obtained on a Bruker Tensor 27 infrared spectrophotometer (Bruker Optics GmbH, Ettlingen, Germany) with KBr pellets. Mass spectra were performed on an API QSTAR time-of-flight spectrometer (MDS Sciqaszex, Concord, Ontario, Canada) and LCMS-IT-TOF (Shimadzu, Kyoto, Japan) spectrometer. NMR spectra were recorded on DRX-500 and Av III-600 instruments with TMS as the internal standard (Bruker, Bremerhaven, Germany). The chemical shifts were given in *δ* (ppm) with reference to the solvent signal. Column chromatography was performed on silica gel (200–300 and 300–400 mesh, Qingdao Marine Chemical Inc., Qingdao, China), Lichroprep RP-18 gel (40–63 μm, Merck, Darmstadt, Germany), MCI gel CHP-20P (75–150 μm, Mitsubishi Chemical Corp., Tokyo, Japan), sephadex LH-20 (20–150 μm, Amersham Biosciences, Uppsala, Sweden), and YMC*GEL ODS-A-HG (50 μm, YMC Co. Ltd. Japan). Fractions were monitored by TLC, and spots were visualized by UV light and sprayed with 10% H_2_SO_4_ in EtOH, followed by heating. 1,1-diphenyl-2-picrylhydrazyl (DPPH), Trolox, mushroom tyrosinase, L-Dopa, and Kojic acid were purchased from Sigma (USA); Transforming growth factor beta (TGF-β) was obtained from Peprotech (USA); Growth media DMEM (high glucose w/L-glut), Hank’s balanced salt solution, fetal bovine serum were purchased from HyClone (USA); Procollagen peptide ELISA kit was obtained from TaKaRa (Japan). All other chemicals and solvents were of analytical grade.

### Plant Material

The stems of *D. loddigesii* were collected in September 2014 from Wenshan City, Yunnan Province, People's Republic of China and identified by Professor Hong Yu (Yunnan University, Kunming, People′s Republic of China). The voucher specimen (No. 20,140,829) has been deposited at the State key Laboratory of Phytochemistry and Plant Resource in West China, Kunming Institute of Botany, Chinese Academy of Sciences.

### Extraction and Isolation

The dried and powdered stems (10.2 kg) of *D. loddigesii* were extracted three times with 80% ethanol under room temperature and concentrated under reduced pressure. Then, the residue was suspended in H_2_O and partitioned with EtOAc to obtain the EtOAc fraction (220 g), which was subjected to silica gel column chromatography eluted with a gradient of petroleum ether/acetone (15:1 to 0:1) to afford 22 fractions (Fr.1–22). Fr.11 (6 g) was subjected to silica gel CC eluted with CHCl_3_/MeOH (300:1), then followed by MCI column (MeOH/H_2_O gradient, 60:40–95:5) and silica gel CC (CHCl_3_/MeOH, 200:1) to yield **12** (4 mg). Fr.13 (850 mg) was separated over a column of MCI (MeOH/H_2_O, 60:40 to 95:5) to give five fractions (Fr.13.1–Fr.13.5). Fr.13.4 (150 mg) afforded compounds **4** (3 mg) and **5** (5 mg) by HPLC preparation (MeOH/H_2_O, 60:40). Fr.16 (19 g) was chromatographed on silica gel column eluted with CHCl_3_/MeOH (100:1 to 20:1) to afford 6 subfractions (Fr.16.1–Fr.16.6). Fr.16.4 (2.3 g) was applied MCI column eluted with MeOH/H_2_O (50:50–100:0) and then further fractionated over a column of sephadex LH-20 (MeOH/H_2_O, 90:10) to yield **7** (716 mg) and **13** (39 mg). By using the same conditions of Fr.16.4, Fr.16.6 (240 mg) afforded compound **9** (7 mg). Fr.18 (10 g) was separated by silica gel CC (CHCl_3_/MeOH, 100:1–20:1), then passed through MCI (MeOH/H_2_O gradient, 50:50–100:0) and sephadex LH-20 (MeOH/H_2_O, 90:10) columns to yield **3** (25 mg) and **11** (4 mg). Fr.19 (20 g) was subjected to MCI column eluted with MeOH/H_2_O (30:70–100:0) to obtain seven fractions (Fr.19.1–Fr.19.7). Fr.19.5 (2.3 g) was separated by repeated silica gel column (CHCl_3_/MeOH, 30:1) to afford **8** (15 mg) and **10** (7 mg). Fr.19.6 (4.3 g) was fractionated on a column of silica gel (CHCl_3_/MeOH, 30:1) to give 5 fractions (Fr.19.6.1– Fr.19.6.5). Fr.19.6.1 (1.2 g) was subjected to silica gel column (CHCl_3_/MeOH, 30:1) and after purification by HPLC (MeOH/H_2_O, 45:55), providing **1** (15 mg). Fr.19.6.3 (540 mg) was applied to a column of sephadex LH-20 column eluted with MeOH/H_2_O (90:10), and then further purified by semipreparative HPLC (MeOH/H_2_O, 45:55) to furnish **2** (6 mg) and **6** (5 mg). Fr.20 (12 g) was applied to a MCI gel (MeOH/H_2_O, 30:70–100:0) chromatographic step and then was subjected to silica gel CC (CHCl_3_/MeOH, 30:1) to give **14** (21 mg).

*threo-*7-*O-*Ethyl*-*9-*O-*(4-hydroxyphenyl)propionyl-guaiacylglycerol (**1**): white solid; [*α*]_D_^26^ – 3.72 (*c* 0.51, MeOH); UV (MeOH) λ_max_ (log *ε*) 203 (4.53), 225 (4.17), 280 (3.66) nm; IR (KBr) *ν*_max_ 3426, 1727, 1516, 829 cm^−1^; ^1^H and ^13^C NMR (CD_3_OD), see Table 1; ESIMS *m/z* 389 [M−H]^−^, HRESIMS *m/z* 389.1602 [M−H]^−^ (calcd. for C_21_H_25_O_7_, 389.1606).

(*R*)-4,5,4′-Trihydroxy-3,3ʹ,α-trimethoxybibenzyl (**2**): white amorphous powder; [*α*]_D_^26^ –12.46 (*c* 1.07, MeOH); UV (MeOH) λ_max_ (log *ε*) 204 (4.59), 286 (3.75) nm; IR (KBr) *ν*_max_ 3418, 1607, 1517, 1455, 1434, 796 cm^−1^; ^1^H and ^13^C NMR (CD_3_OD), see Table 1; ESIMS *m/z* 319 [M−H]^−^, HRESIMS *m/z* 319.1180 [M−H]^−^ (calcd. for C_17_H_19_O_6_, 319.1187).

(2*S*)-5,7,3ʹ-Trihydroxy-6,4,5-trimethoxyflavone (**3**): yellow amorphous powder; [*α*]_D_^26^ – 46.64 (*c* 0.46, MeOH); UV (MeOH) λ_max_ (log *ε*) 206 (4.70), 294 (4.17) nm; IR (KBr) *ν*_max_ 3335, 2940, 1641, 1514, 1462, 1345, 1434, 1182, 1091, 998, 833 cm^−1^; ^1^H and ^13^C NMR (CD_3_OD), see Table 1; ESIMS *m/z* 331 [M−H]^−^, HRESIMS *m/z* 331.0819 [M−H]^−^ (calcd. for C_17_H_15_O_7_, 331.0823).

### DPPH Radical Scavenging Activity Assay

The free radical scavenging activity assay was carried out according to previous method [36] with some modifications. Briefly, 30 μL samples (1000 μg/mL, dissolved in ethanol) and Trolox (1 mM) were added to 270 μL DPPH solution (100 μM, dissolved in methanol), respectively. The reaction proceeded for 1 h at 37 °C on a 96-well microplate. The absorbance was then read at 515 nm and percentage of total radical scavenging activity was calculated using the following formula: inhibition % = [(A_0_ − A_1_)/A_0_] × 100%, where A_0_ is the absorbance of the DPPH without samples (control reaction) and A_1_ is the absorbance of DPPH incubated with the samples. All the tests were conducted in triplicate and Trolox was used as a positive control agent.

### Mushroom Tyrosinase Inhibitory Assay

Tyrosinase activity inhibition was determined spectrophotometrically according to the method described previously [36] with some modifications. Briefly, different concentrations of test compounds were prepared in 10% DMSO. Each of the sample solution (20 μM) were mixed with L-Dopa (1.25 mM), and diluted with 970 *μ*L of 0.05 mM sodium phosphate buffer (PBS, pH 6.8) in the test tubes. The reaction was initiated by adding mushroom tyrosinase (25 U/mL). The reaction mixture was incubated for 5 min at room temperature. The amount of Dopachrome in the mixture was determined by the measurement of the absorbance of each well at 490 nm. Kojic acid was used as positive control. The inhibitory percentage of tyrosinase was calculated according to the following equation: Percent inhibition = [(A_0_ − A_1_)/A_0_] × 100%, where A_0_ is the absorbance of the Dopachrome without test compounds (control reaction) and A_1_ is the absorbance of Dopachrome incubated with the test compounds.

### Collagen Production by HDFa Assay

The HDFa cell line was obtained from Cascade Biologics. HDFa cells were seeded in 96-well plates containing DMEM with 10% FBS under a humidified atmosphere of 5% CO_2_ at 37 °C. After 24 h of incubation, the cells were treated with the test samples for 72 h (37 °C, 5% CO_2_). TGF-β was used as the positive control. Media (50 µL) was collected from each well, and froze at − 80 °C until it was assayed with procollagen peptide ELISA kit. The concentration of pro-collagen was obtained by measuring the absorbance at 450 nm on the microplate reader. Remove all media from cells and add 100 µL diluted MTS reagent to each well. The reaction incubated for 40 min at 37 °C. The absorbance was measured at 490 nm with a microplate reader. The increase percentage of collagen I production was calculated according to the following equation: cell viability (%) = (Mean OD_490_ sample/Mean OD_490_ control); increase of collagen production % = (A_1_/B/A_0_ − 1) × 100%. Where A_1_ is the absorbance with the samples, A_0_ is the absorbance without samples (control reaction), and B is cell viability.

## Electronic supplementary material

Below is the link to the electronic supplementary material.

Supplementary file1 (DOCX 18883 kb). Supplementary material (1H-, 13C NMR, DEPT, HSQC, HMBC, COSY, HRESIMS, IR, UV spectra of compounds 1–3) is available in the online version of this article and is accessible for authorized users.
