# Supplementary material for: Phenolic Constituents with Antioxidative, Tyrosinase Inhibitory and Anti-aging Activities from Dendrobium loddigesii Rolfe
Source: Nat Prod Bioprospect. 2019 Oct 19;9(5):329–36. doi: 10.1007/s13659-019-00219-y (PMC6814690; doi:10.1007/s13659-019-00219-y)
Supplement: Supplementary file 1 — Supplementary file1 (DOCX 18883 kb). Supplementary material (1H-, 13C NMR, DEPT, HSQC, HMBC, COSY, HRESIMS, IR, UV spectra of compounds 1–3) is available in the online version of this article and is accessible for authorized users. [file 13659_2019_219_MOESM1_ESM.docx]

**Phenolic constituents, antioxidative,** **tyrosinase inhibitory and anti-aging activities from** ***Dendrobium loddigesii*** **Rolfe**

**Rui-Jing Ma^a,b,†^ ∙ Liu Yang^a,c,†^ ∙ Xue Bai^a^ ∙ Jin-Yu Li^a^ ∙ Ming-Yan Yuan^a,c^ ∙ Ya-Qin Wang^c^ ∙ Yong Xie^c^ ∙ Jiang-Miao Hu^a,c,^* ∙ Jun Zhou^a^**

^a^ State Key Laboratory of Phytochemistry and Plant Resources in West China, Kunming Institute of Botany, Chinese Academy of Sciences, Kunming 650201, People′s Republic of China

^b^ College of pharmacy, Guilin Medical University, Guilin 541004, People′s Republic of China

^c^ R & D Center of Dr. Plant, Kunming Institute of Botany, Chinese Academy of Sciences, Kunming 650201, People′s Republic of China

^†^ These authors contribute equally to this work.

Corresponding Author:

***** Tel: (86) 871-65223264. E-mail: [hujiangmiao@mail.kib.ac.cn](mailto:hujiangmiao@mail.kib.ac.cn)

**Supporting Materials**

|  | **Pag.** |
| --- | --- |
| Figure S1 ^1^H NMR spectrum of compound **1** | 3 |
| Figure S2 ^13^C NMR spectrum of compound **1** | 4 |
| Figure S3 HSQC spectrum of compound **1** | 5 |
| Figure S4 HMBC spectrum of compound **1** | 6 |
| Figure S5 COSY spectrum of compound **1** | 7 |
| Figure S6 HRESIMS spectrum of compound **1** | 8 |
| Figure S7 IR spectrum of compound **1** | 9 |
| Figure S8 UV spectrum of compound **1** | 10 |
| Figure S9 ^1^H NMR spectrum of compound **2** | 11 |
| Figure S10 ^13^C NMR spectrum of compound **2** | 12 |
| Figure S11 HSQC spectrum of compound **2** | 13 |
| Figure S12 HMBC spectrum of compound **2** | 14 |
| Figure S13 COSY spectrum of compound **2** | 15 |
| Figure S14 HRESIMS spectrum of compound **2** | 16 |
| Figure S15 IR spectrum of compound **2** | 17 |
| Figure S16 UV spectrum of compound **2** | 18 |
| Figure S17 ^1^H NMR spectrum of compound **3** | 19 |
| Figure S18 ^13^C NMR spectrum of compound **3** | 20 |
| Figure S19 HSQC spectrum of compound **3** | 21 |
| Figure S20 HMBC spectrum of compound **3** | 22 |
| Figure S21 COSY spectrum of compound **3** | 23 |
| Figure S22 HRESIMS spectrum of compound **3** | 24 |
| Figure S23 IR spectrum of compound **3** | 25 |
| Figure S24 UV spectrum of compound **3** | 26 |

Figure S1. ^1^H NMR spectrum (CD_3_OD, 500 MHz) of **1**

Figure S2. ^13^C NMR spectrum (CD_3_OD, 500 MHz) of **1**

Figure S3. HSQC spectrum (CD_3_OD, 500 MHz) of **1**

Figure S4. HMBC spectrum (CD_3_OD, 500 MHz) of **1**

Figure S5. COSY spectrum (CD_3_OD, 500 MHz) of **1**

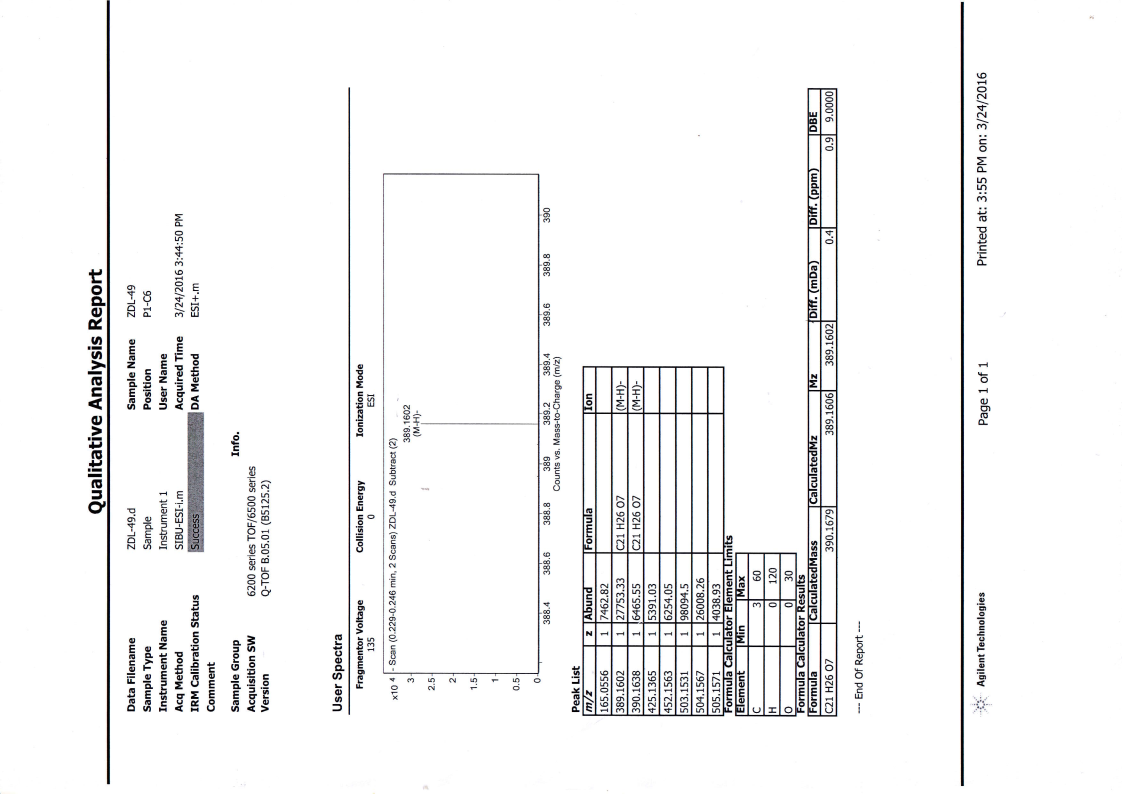


Figure S6. HR-ESI-MS spectrum **1**


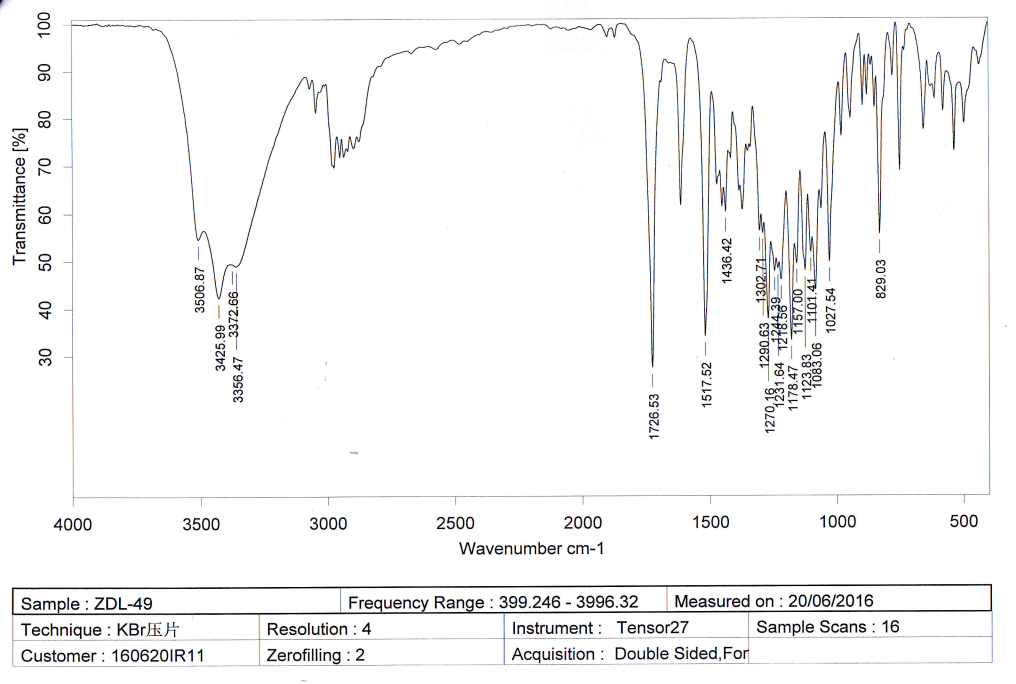


Figure S7. IR spectrum **1**


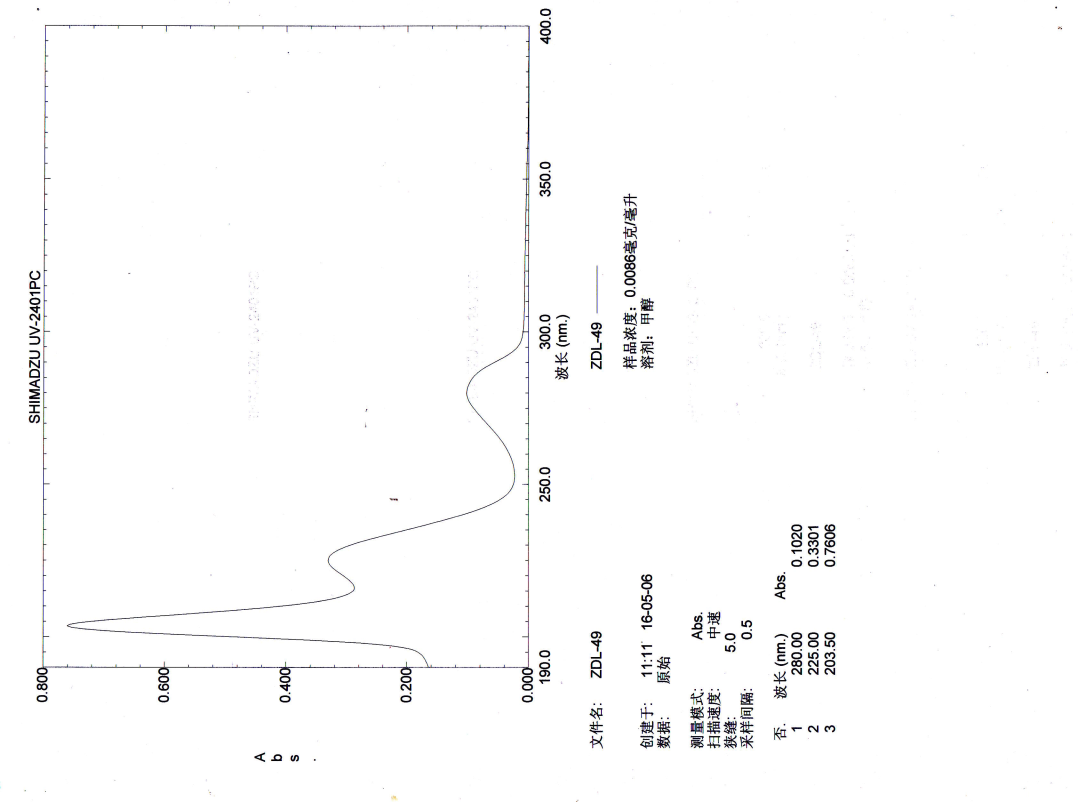


Figure S8. UV spectrum of **1**

Figure S9. ^1^H NMR spectrum (CD_3_OD, 600 MHz) of **2**

Figure S10. ^13^C NMR spectrum (CD_3_OD, 600 MHz) of **2**

Figure S11. HSQC spectrum (CD_3_OD, 600 MHz) of **2**

Figure S12. HMBC spectrum (CD_3_OD, 600 MHz) of **2**

Figure S13. COSY spectrum (CD_3_OD, 600 MHz) of **2**


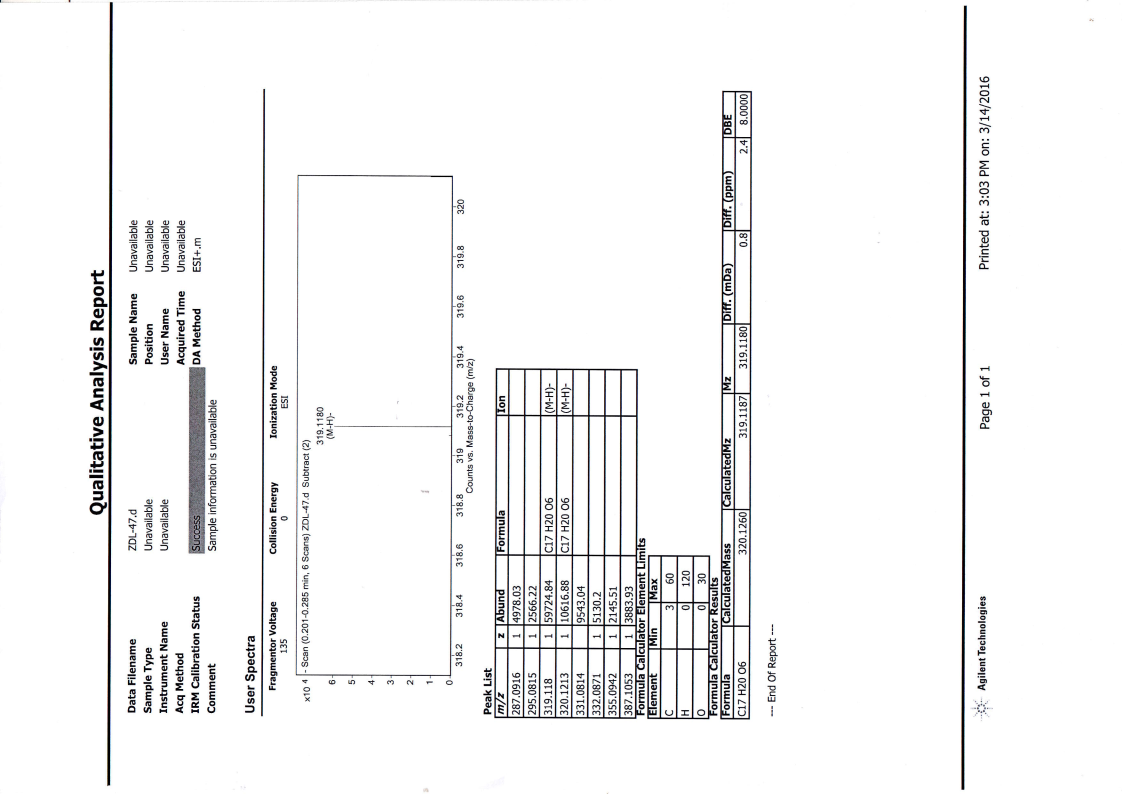


Figure S14. HR-ESI-MS spectrum of **2**


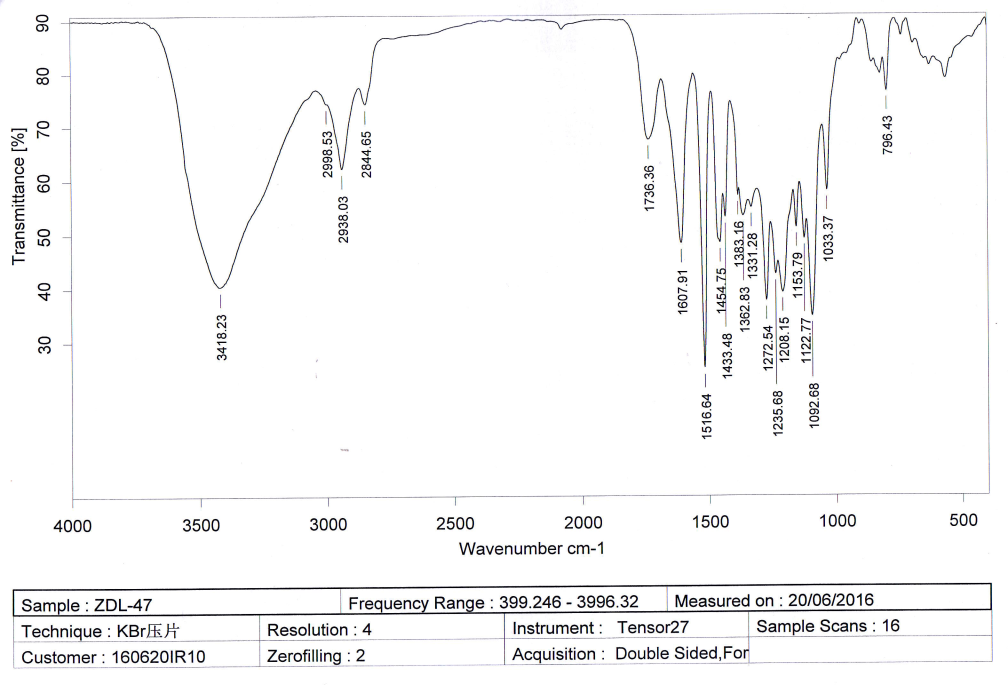


Figure S15. IR spectrum of **2**


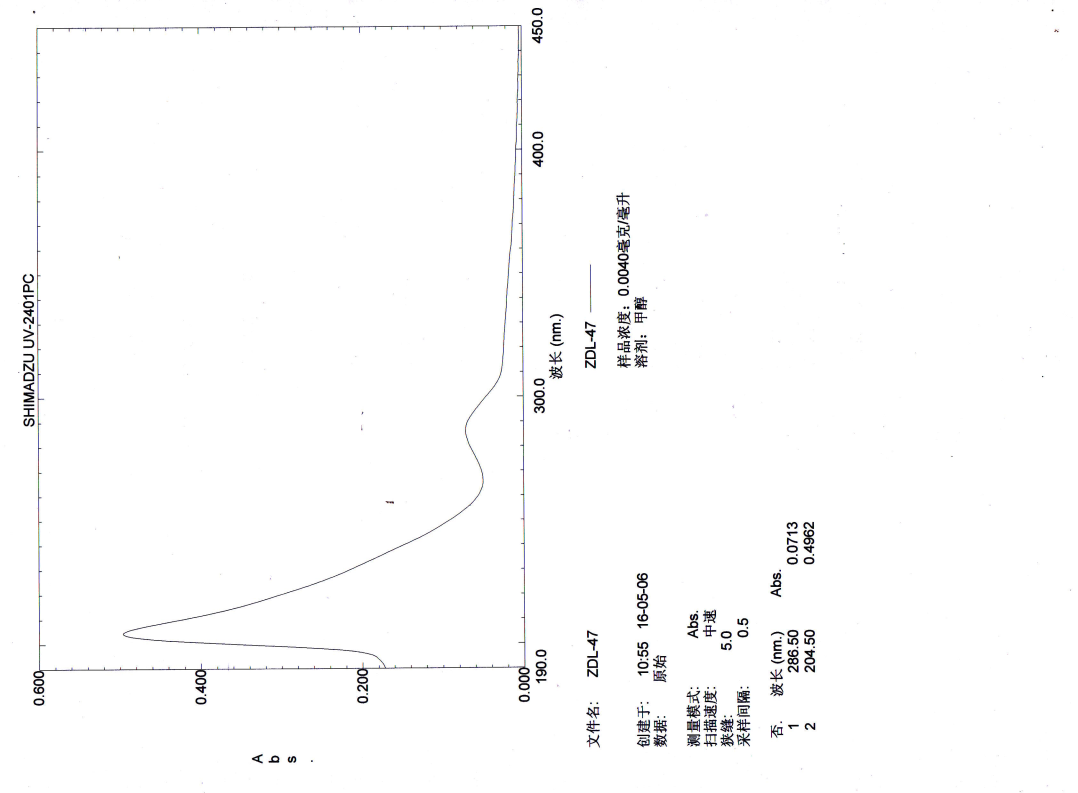


Figure S16. UV spectrum of **2**

Figure S17. ^1^H NMR spectrum (CD_3_OD, 600 MHz) of

Figure S18. ^13^C NMR spectrum (CD_3_OD, 600 MHz) of **3**

Figure S19. HSQC spectrum (CD_3_OD, 600 MHz) of **3**

Figure S20. HMBC spectrum (CD_3_OD, 600 MHz) of **3**

Figure S21. COSY spectrum (CD_3_OD, 600 MHz) of **3**


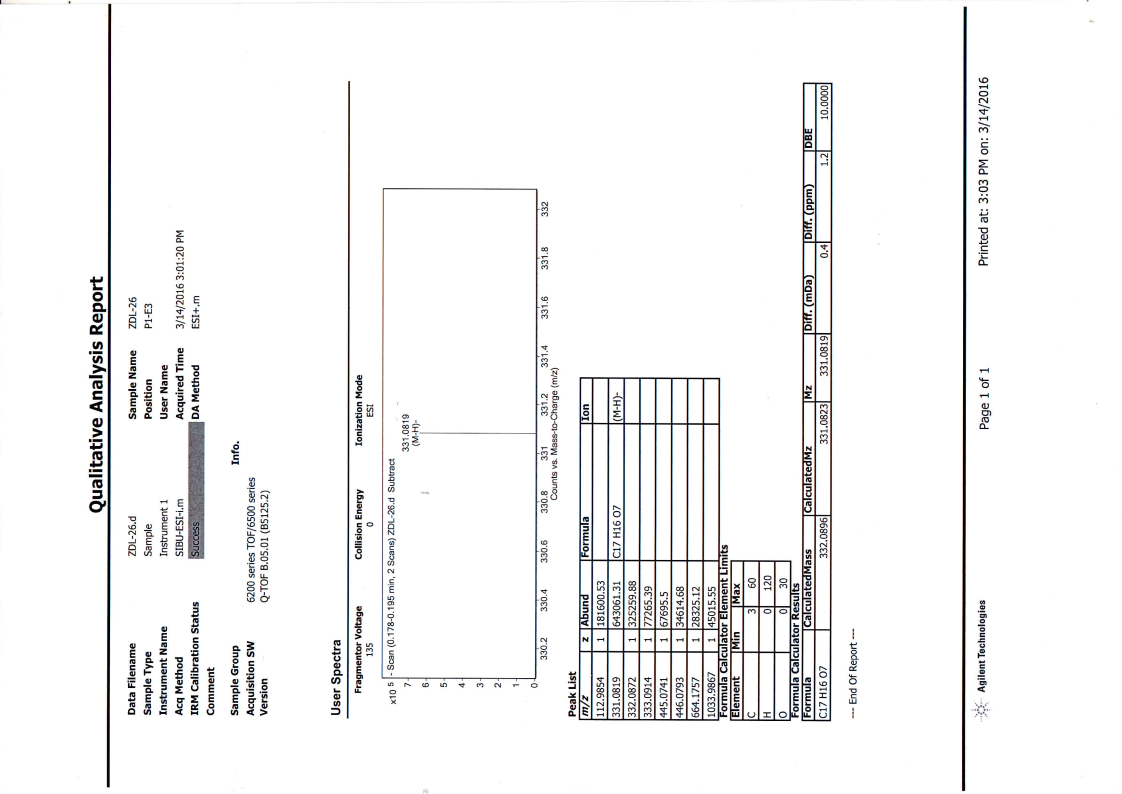


Figure S22. HR-ESI-MS spectrum of **3**


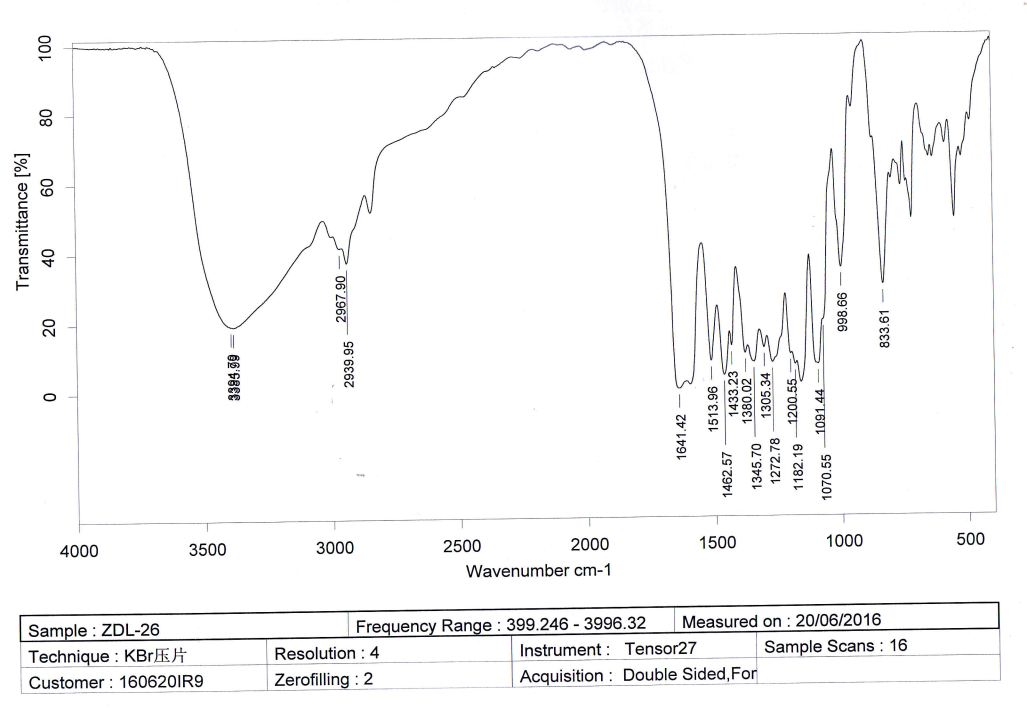


Figure S23. IR spectrum of **3**


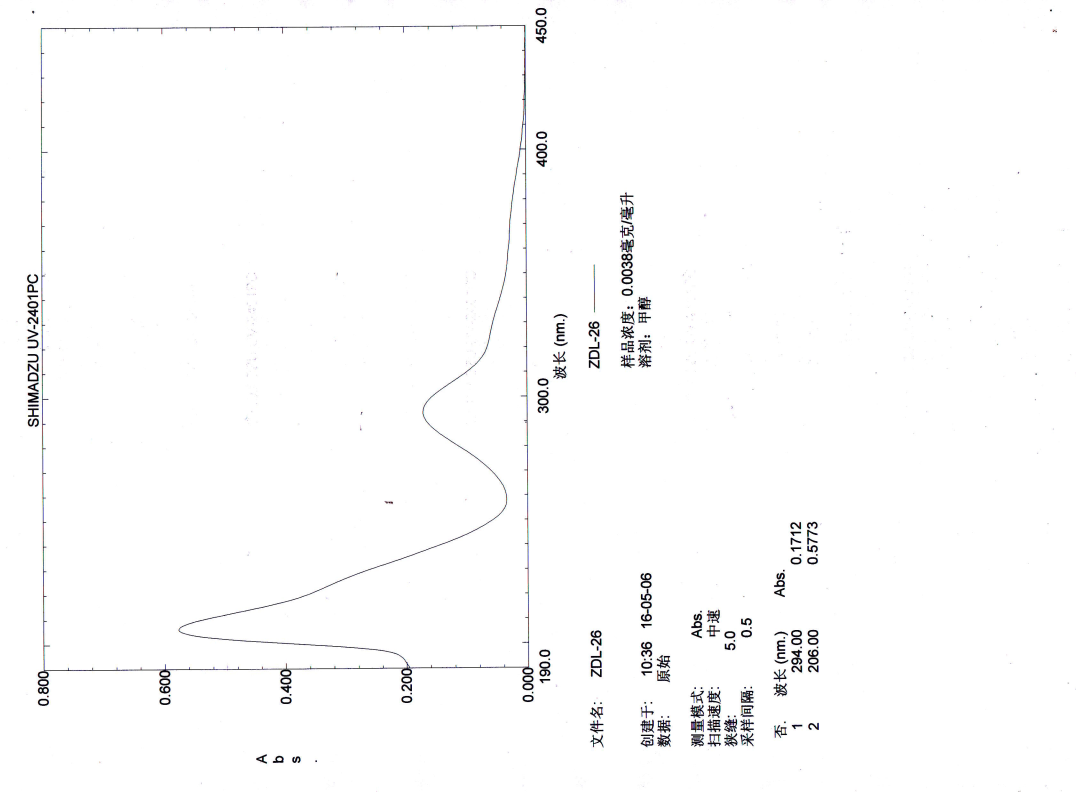


Figure S24. UV spectrum of **3**
